# Alpinumisoflavone Exhibits the Therapeutic Effect on Prostate Cancer Cells by Repressing AR and Co-Targeting FASN- and HMGCR-Mediated Lipid and Cholesterol Biosynthesis

**DOI:** 10.3390/life12111769

**Published:** 2022-11-02

**Authors:** Praveenkumar Basavaraj, Phakkhathorn Ruangsai, Po-Fan Hsieh, Wen-Ping Jiang, Da-Tian Bau, Guan-Jhong Huang, Wen-Chin Huang

**Affiliations:** 1 Graduate Institute of Biomedical Sciences, School of Medicine, China Medical University, Taichung 404, Taiwan; 2 International Master’s Program of Biomedical Sciences, School of Medicine, China Medical University, Taichung 404, Taiwan; 3 Department of Urology, China Medical University Hospital, Taichung 404, Taiwan; 4 Department of Pharmacy, Chia Nan University of Pharmacy and Science, Tainan 717, Taiwan; 5 Terry Fox Cancer Research Laboratory, Department of Medical Research, China Medical University Hospital, Taichung 404, Taiwan; 6 Department of Bioinformatics and Medical Engineering, Asia University, Taichung 404, Taiwan; 7 School of Chinese Pharmaceutical Sciences and Chinese Medicine Resources, College of Chinese Medicine, China Medical University, Taichung 404, Taiwan

**Keywords:** alpinumisoflavone, androgen receptor, anti-prostate cancer efficacy, apoptosis, fatty acid synthase, 3-hydroxy-3-methylglutaryl-CoA reductase, lipid and cholesterol biosynthesis

## Abstract

Prostate cancer (PCa) is the most common cancer in men, and this has been mainly noticed in Western and Asian countries. The aggregations of PCa and castration-resistant PCa (CRPC) progression are the crucial causes in the mortality of patients without the effective treatment. To seek new remedies for the lethal PCa diseases is currently an urgent need. In this study, we endeavored to investigate the therapeutic efficacy of alpinumisoflavone (AIF), a natural product, in PCa. LNCaP (androgen- sensitive) and C4-2 (CRPC) PCa cells were used. An MTT-based method, soft agar colony forming assay, biological progression approaches were applied to determine cell viability, migration, and invasion. A fatty acid quantification kit, a cholesterol detection kit and oil red O staining were conducted to analyze the intracellular levels of lipids and cholesterols. Apoptosis assays were also performed. AIF reduced cell viability, migration, and invasion in PCa cells. The expression of androgen receptor (AR), fatty acid synthase (FASN), and 3-hydroxy-3-methylglutaryl-CoA reductase (HMGCR) was substantially inhibited by AIF treatment in PCa cells. Furthermore, by inhibiting FASN and HMGCR expression, AIF decreased the amounts of intracellular fatty acids, cholesterols, and lipid droplets in PCa cells. Significantly, through coordinated targeting FASN- and HMGCR-regulated biosynthesis and the AR axis, AIF activated the caspase-associated apoptosis in PCa cells. These results collectively demonstrated for the first time the potential of AIF as a novel and attractive remedy and provided an alternative opportunity to cure PCa malignancy.

## 1. Introduction

Natural products essentially exhibit the critical bio-functions for the therapy of numerous diseases. The advantages of natural products evidently display distinctive characters when compared with regular synthetic molecules [1]. Alpinumisoflavone (AIF) is a natural product compound showing several important bioactivities and is originally extracted from *Derris eriocarpa*, a traditional Chinese medicinal herb [2], or isolated from mandarin melon berry (*Cudrania tricuspidata* Bur. ex Lavallee) [3]. Significantly, AIF displayed potentially antiestrogenic activity in breast cancer and attenuated the osteoclast differentiation as well as induced osteocytes apoptosis [4,5]. However, the therapeutic effect and the molecular basis of AIF in prostate cancer (PCa) remain to be explored.

PCa is the most regular type of cancer diagnosed in men [6]. Additionally, PCa cells are specifically in need of androgens and the androgen receptor (AR) pathway for survival, growth, and even aggressiveness [7]. An advanced form of progression and metastatic disease of PCa succeeding pharmaceutical castration, particularly with androgen deprivation therapy (ADT), eventually showed a result of cancer recurrence and aggressiveness from the status of hormone-naïve to lethal castration-resistant prostate cancer (CRPC). The deadly CRPC progression remains the most critical and challenge issue clinically and the very limited therapeutic options in the current [8].

The alteration of metabolic activity in cancer cells is literally considered to be one of the main hallmarks of cancer [9], and contributes to the induction of unrestrained cell growth as well as the oncogenic signaling pathways as the ordinary cancerous phenotypes [10]. In the PCa study, the activation of lipogenesis and cholesterogenesis profoundly affected cancer cell growth and CRPC progression [11,12]. Among the metabolic regulatory factors, fatty acid synthase (FASN) and 3-hydroxy-3-methylglutaryl-CoA reductase (HMGCR) are key and rate-limiting regulators to activate de novo biosynthesis of fatty acids/lipids (lipogenesis) and cholesterols (cholesterogenesis), respectively [13,14], and further provide the fundamental components of cell membranes supporting rapid cancer cell growth and inducing the survival signal transduction [15,16,17]. Significantly, the clinical data revealed that FASN and HMGCR proteins were greatly expressed in advanced PCa patients’ tumor specimens, including CRPC, compared to the normal prostate tissue samples [17,18]. It is noteworthy that suppression of FASN and/or HMGCR expression by small synthetic molecules has been demonstrated to inhibit cell growth and metastasis as well as induce cell death in PCa malignancy and CRPC [19,20,21]. Therefore, targeting the key metabolic regulators specifically for lipogenesis and cholesterogenesis would be able to offer a novel and alternative therapeutic direction toward the treatment of PCa aggressiveness, including CRPC.

In this study, we evaluated the therapeutic significances and determined the molecular features of AIF in various clinically relevant PCa cell lines, LNCaP (androgen-sensitive) and C4-2 (CRPC) cells. AIF substantially suppressed cell growth, colony formation, migration, and invasion showing a concentration-dependent pattern in both LNCaP and C4-2 cells. Furthermore, we investigated the capability of AIF on the expression of key metabolic and signaling regulators, including FASN, HMGCR, AR, and prostate-specific antigen (PSA), which are beneficial for PCa development and progression. Through downregulation of the metabolic gene expression, AIF lowered the amounts of fatty acids and cholesterols, and reduced the intracellular lipid droplets in PCa cells. Moreover, AIF treatment led to cell death via activation of the caspase-associated apoptotic pathway in PCa cells. Collectively, these results provided the evidence for the first time that AIF could be used as a promising remedy toward the treatment of aggressive PCa.

## 2. Materials and Methods

### 2.1. Preparation of AIF

AIF (Supplementary Figure S1) was purchased from BJYM Pharm. & Chem. Co. Ltd. (Beijing, China). Subsequently, AIF powder was dissolved in dimethyl sulfoxide (DMSO; Sigma-Aldrich Chemicals, St. Louis, MO, USA) as a stock solution and storage at −20 °C until use.

### 2.2. PCa Cell Lines and Culture Condition

The human PCa cell lines [22], androgen-sensitive (LNCaP), and CRPC (C4-2) cells, were supported by Dr. Leland W.K. Chung (Cedars-Sinai Medical Center, Los Angeles, CA, USA). Cells were cultured in RPMI 1640 medium (Thermo Fisher Scientific/GIBCO, Waltham, MA, USA) supplemented with 10% fetal bovine serum (GE Healthcare/Hyclone, Pittsburgh, PA, USA), 100 U/mL penicillin, and 100 μg/mL streptomycin at 37°C with a humidified incubator containing 5% CO_2_.

### 2.3. Cell Growth Analysis

To investigate the therapeutic effects of AIF on cell growth, PCa cells were seeded in the 96-well plates (1 × 10^4^ cells/well) and incubated overnight. Subsequently, cells were treated with disparate concentrations of AIF (10, 20, 40, 80, 100, and 120 μM) or vehicle control (1.2% DMSO; this concentration would not change the biological behaviors of PCa cells) for 24 and 48 h. Cell growth was verified by MTT assay (Promega, Madison, WI, USA) in accordance with the manufacturer’s instructions [23]. In addition, PCa cell viability was assayed by soft agar colony formation assay. PCa cells were seeded in a 0.3% agarose gel mixed with culture medium and the various concentrations of AIF or vehicle control using the 6-well plates [24]. These plates were incubated with 5% CO_2_ at 37 ℃ for 25 days. The colonies of LNCaP and C4-2 were observed under an inverted microscope and calculated the averages of the colony numbers and the sizes by an ImageJ software (ij153-win-java8).

### 2.4. Cell Migration and Invasion Analyses

The capability of cell movement was measured by a wound healing analysis. Briefly, PCa cells were grown in 6-well cell culture plates and incubated until reaching confluency. A straight artificial scratch was created by a P200 micropipette tip. The cells were then treated with the various concentrations of AIF or control (1.2% DMSO). Cell spread across the wound was recorded under a light microscope equipped with a camera at 24, 48, and 96 h. Furthermore, the migration and invasion assays of PCa cells were performed utilizing the 24-well culture plates with Transwell chambers [25]. The membranes of the Transwell chamber were firstly coated with nothing (migration assay) or the growth factor reduced Matrigel basement membrane matrix (Corning, Bedford, MA, USA) (invasion assay). Subsequently, LNCaP and C4-2 cells (5 × 10^5^ cells/well) were seeded in the chamber and then treated with AIF or control (1.2% DMSO). After 48 h treatment, the migrated or invaded cells were fixed with 100% cold methanol and stained with a crystal violet (0.005%) solution. After dehydration, the inverted microscope was used to count the numbers of PCa cells in 3-randomly selected regions of the migrated or invaded cells.

### 2.5. Quantitative Reverse Transcription-Polymerase Chain Reaction (RT-qPCR)

Total RNA samples were extracted from the AIF or control-treated PCa cells using REzolTM C&T (Protech Technology, Taipei, Taiwan). Next, cDNA was converted by utilizing an iScriptTM cDNA Synthesis Kit (Bio-Rad, Hercules, CA, USA). The qPCR sample preparation was executed by applying iQ SYBR Green Supermix (Bio-Rad) with the specific DNA sequences of oligo-primers including AR, PSA, FASN, HMGCR, and β-actin (Table S1). The qPCR analytical procedure was performed with CFX96 Touch Real-Time PCR System (Bio-Rad). Data were shown as the average ratio of triplicates and normalized to internal control β-actin.

### 2.6. Western Blot Analysis

Total protein samples were prepared from LNCaP and C4-2 cells treated with AIF or control by RIPA Lysis Buffer containing protease inhibitors. The amounts of proteins were determined by a PierceTM BCA Protein Assay Kit (Thermo Fisher Scientific). The equal amounts of the protein samples were loaded in SDS-PAGE gel for electrophoresis [26]. After electrophoresis, the separated proteins were transferred into polyvinylidene difluoride (PVDF) membranes and blocked by 5% reconstituted skim milk in PBS with Tween-20. Subsequently, the blotted membranes were incubated with primary antibodies for overnight. The detailed information of the used primary antibodies in this study as follow: anti-AR, anti-FASN (Santa Cruz Biotechnology, Dallas, TX, USA), anti-HMGCR (Abcam, Cambridge, UK), anti-BAX (Cell Signaling Technology, Danvers, MA, USA), anti-caspase-3 (Novus Biologicals, Centennial, CO, USA), anti-PARP, and anti-β-actin (GeneTex, Irvin, CA, USA). Next, these membranes were incubated with the secondary antibody. The visualization of the specific protein signal was detected by Enhanced Chemiluminescence Reagent kit (Amersham Biosciences, Arlington Heights, IL, USA) with the ImageQuantTM LAS 4000 system. Finally, the quantification of the protein band was analyzed by an ImageJ software (ij153-win-java8) with normalizing β-actin protein.

### 2.7. Lipogenesis and Cholesterogenesis Assays

For the measurement of fatty acids and cholesterols in PCa cells, cells were treated with AIF or control and subsequently examined both the intracellular levels of fatty acids by a Free Fatty Acid Quantification kit (MBL International Corporation, Woburn, MA, USA) and the levels of cholesterols by filipin staining (Abcam) according to the manufacturer’s instructions. Additionally, for the assessment of lipid droplet accumulation, an Oil Red O staining assay was performed [27]. Bright field microscopy was utilized to visualize and record the positively stained cells treated with AIF or control. And for the quantification of the levels of lipid droplets, Oil Red O in cells were extracted by isopropanol and determined by the absorbance at 500 nm. Results were normalized by the numbers of total cells for each assay.

### 2.8. Apoptosis Analysis

PCa cells were exposed to AIF or control for 48 h. The treated cells were subsequently assayed by Annexin V-FITC and propidium iodide (PI) Detection Kit (Biolegend, San Diego, CA, USA) based on the manufacturer’s instruction. Flow cytometers with an FCS Express v2.0 software (BD FACSCanto, BD Bioscience, Franklin Lakes, NJ, USA) were applied to and analyzed by the apoptotic cells (%).

### 2.9. Statistical Analysis

The independent sample *t*-test, statistical hypothesis testing, was performed to analyze the comparison of all of the data representation at least three individual experiments. Data were indicated as the mean ± SD. *p* values less than 0.05 were considered to be the statistical significance.

## 3. Results

### 3.1. AIF Suppressed PCa Cell Growth, Colony Formation, Migration, and Invasion

**Figure 1:**
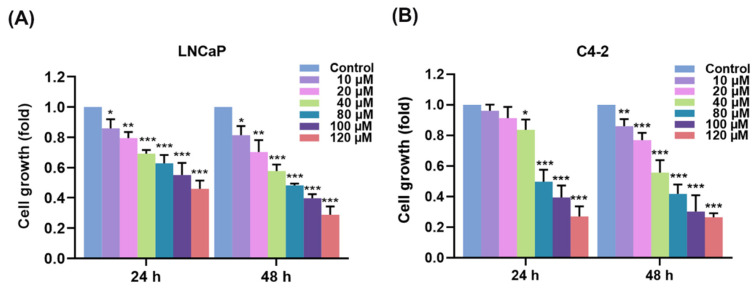
AIF suppressed PCa cell growth. (**A**) LNCaP and (**B**) C4-2 cells were exposed to AIF at different concentrations or vehicle control for 24 and 48 h. MTT assay was utilized to determine cell growth. The relative cell growth (fold) was represented as 1.0 in control-medicated PCa cells at 24 or 48 h. Data indicated as the mean ± SD of three independent experiments. (*) *p* < 0.05, (**) *p* < 0.01, (***) *p* < 0.001.

To investigate the essential effects of AIF on PCa cells with clinical relevance, we conducted several bio-functional experiments, including cell growth, soft agar colony formation, migration, and invasion. LNCaP and C4-2 cells were utilized in this study. For cell growth assay, PCa cells were treated with different doses of AIF and vehicle control (1.2% DMSO) for 24 and 48 h. Subsequently, MTT assay was performed to evaluate cell growth. As shown in Figure 1A,B, AIF significantly suppressed cell growth in both LNCaP and C4-2 cells with a dose-dependent pattern.

**Figure 2:**
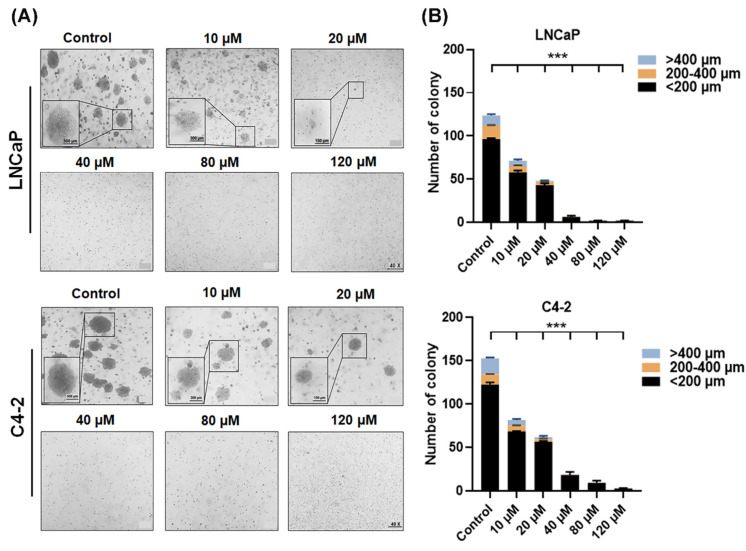
AIF inhibited anchorage-independent growth in PCa cells. Soft agar colony formation assay was performed in (**A**) AIF or control (1.2% DMSO) were used to treat LNCaP and C4-2 cells for 25 days. The characterization images of colony formation were shown. (**B**) The quantified data with the different colony sizes were shown. (***) *p* < 0.001.

Furthermore, an anchorage-independent growth analysis for the measurement of the ability of cells to proliferate into 3-D semi-solid matrices in a soft agar colony formation assay [28], was utilized. The results showed a direct correlation between the numbers as well as the sizes of colonies formed in the soft agar and the doses of AIF treatment in LNCaP and C4-2 cells (Figure 2).

**Figure 3:**
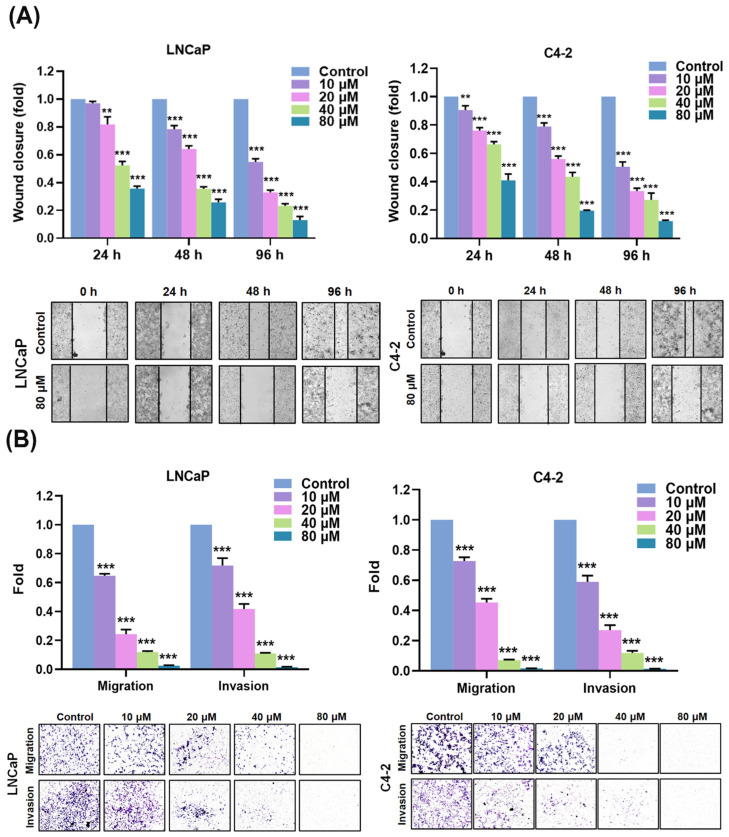
AIF attenuated PCa cell migration and invasion. (**A**) A wound healing assay was performed to determine cell migration in LNCaP and C4-2 cells treated with control or AIF. The analyses of wound closure were represented at 24, 48, and 96 h, respectively. The quantification of wound closure (the top panels) and the representative images of wound closure (the bottom panels) were shown. (**B**) Transwell migration and invasion assays were defined the migration and invasion in both LNCaP and C4-2 cells in accordance with AIF treatment and control for 48 h. The relative migration and invasion were assigned as 1.0 (Fold) in the control-treated cells. The quantification of results (the top panels) and the representative images of Transwell migration and invasion (the bottom panels) were shown. The data is indicated as three different studies. (**) *p* < 0.01, (***) *p* < 0.001.

Moreover, the influences of AIF in migration as well as invasion of PCa cells were examined by a wound healing assay and the Transwell methods. The data of a wound healing assay demonstrated the suppression of wound closure by AIF treatment with a dose- and a time-dependent manner in both LNCaP and C4-2 cells (Figure 3A). Similarly, the results of the Transwell methods showed that AIF significantly affected the potentials of PCa cell migration and invasion in a concentration-dependent inhibition (Figure 3B). Taken together, the data of the bio-functional analyses confirmed that AIF displayed the effects to suppress PCa cell growth, the numbers and the sizes of colony formation, migration, and invasion.

### 3.2. AIF Inhibited AR and PSA as Well as FASN and HMGCR Expression in PCa Cells

**Figure 4:**
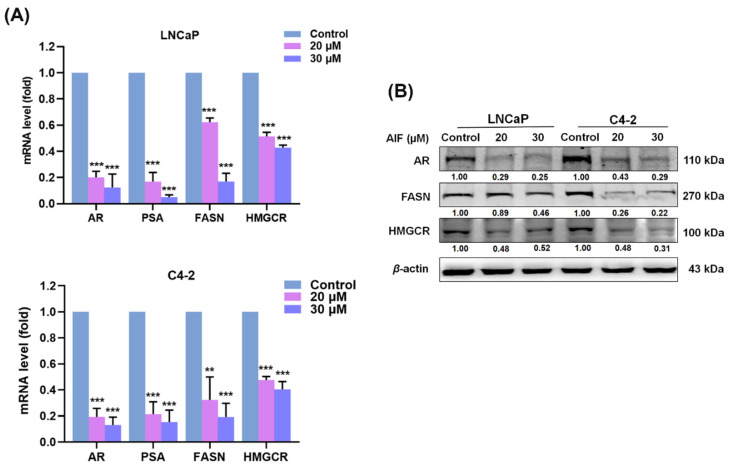
AIF inhibited AR, PSA, FASN and HMGCR in PCa cells. (**A**) The mRNA expression levels of AR, PSA, FASN, and HMGCR were reduced by AIF treatment compared to control in both LNCaP and C4-2 cells. The relative mRNA level (fold) was assigned as 1.0 in the control-treated cells. (**B**) The protein expression levels of AR, FASN, and HMGCR were inhibited by AIF treatment. The relative protein level (fold) was defined as 1.00 in the control-treated cells for each cell lines. Data indicated as the mean ± SD of triplicate assays in three independent experiments and were normalized by β-actin expression. (**) *p* < 0.01, (***) *p* < 0.001.

The previous studies reported that one of the functions of androgen/AR could be correlated with the metabolic pathway, including lipid and cholesterol biosynthesis in PCa cells [29,30,31]. Additionally, by targeting the AR signaling and diminishing FASN expression, PCa cells led to inhibition of cell growth, migration, and invasion [32]. To explore the possible molecular basis by which AIF suppressed cell growth, migration, and invasion, the expression of key genes related to the AR axis, lipogenesis, and cholesterogenesis were evaluated by RT-qPCR and Western blot analyses. As shown in Figure 4A, AIF greatly reduced the mRNA levels of AR and PSA compared to the control group in both LNCaP and C4-2 cells. Particularly, the mRNA expression levels of FASN and HMGCR were also inhibited by AIF in PCa cells (Figure 4A). Moreover, the results of the Western blot analysis showed that the relative levels of AR, FASN, and HMGCR proteins were decreased by AIF treatment in PCa cells (Figure 4B). Collectively, our data revealed the potential molecular mechanism that AIF inhibited the gene expression of AR/PSA, FASN, and HMGCR to further block the AR-associated signaling axis, as well as metabolic lipogenesis and cholesterogenesis in LNCaP and C4-2 PCa cells.

### 3.3. AIF Decreased the Fatty Acid Levels, the Cholesterol Amounts and Lipid Droplet Accumulation in PCa Cells

**Figure 5:**
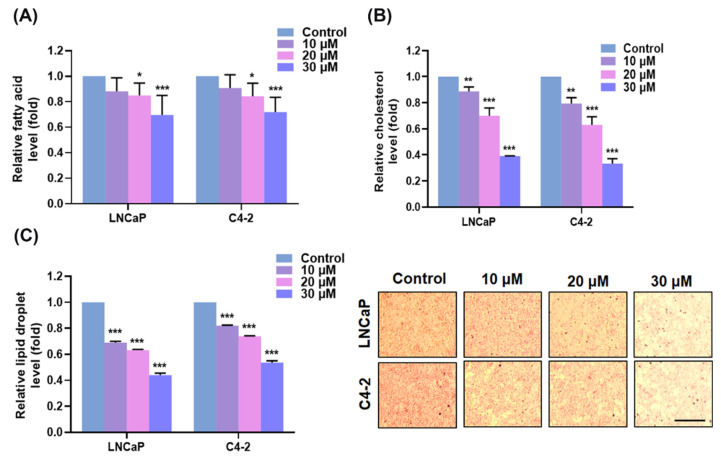
AIF decrased fatty acid, cholesterol and lipid biosynthesis in PCa cells. (**A**) The levels of intracellular fatty acids were suppressed by AIF treatment compared to the control group in LNCaP and C4-2 cells. (**B**) The levels of cholesterols were significantly reduced by AIF in LNCaP and C4-2 cells. (**C**) AIF treatment decreased lipid droplet accumulation determined by an Oil Red O assay in PCa cells (the left panel). The representative images of lipid droplet accumulation in LNCaP and C4-2 cells were shown (the right panel). Scale bar = 100 μm. The data of quantification (fold) were normalized by cell numbers. (*) *p* < 0.05, (**) *p* < 0.01, (***) *p* < 0.001.

We next investigated if AIF altered the intracellular fatty acid, the cholesterol levels and lipid droplet accumulation through inhibition of FASN and HMGCR expression in PCa cells determined by the commercially available kits and an Oil Red O staining method. As shown in Figure 5A, AIF decreased the relative levels of fatty acids in both LNCaP and C4-2 cells with a dose-dependent pattern compared to the control group. In addition, the intracellular cholesterol amounts were also reduced by AIF treatment in PCa cells (Figure 5B). As expected, AIF significantly affected lipid droplet accumulation in PCa cells with a concentration-dependent reducing manner (Figure 5C). Taken together, these results of the biological assays indicated that lipogenesis and cholesterogenesis were attenuated by AIF in PCa cells.

### 3.4. AIF Induced PCa Cell Death via Activation of Caspase-Dependent Apoptosis

**Figure 6:**
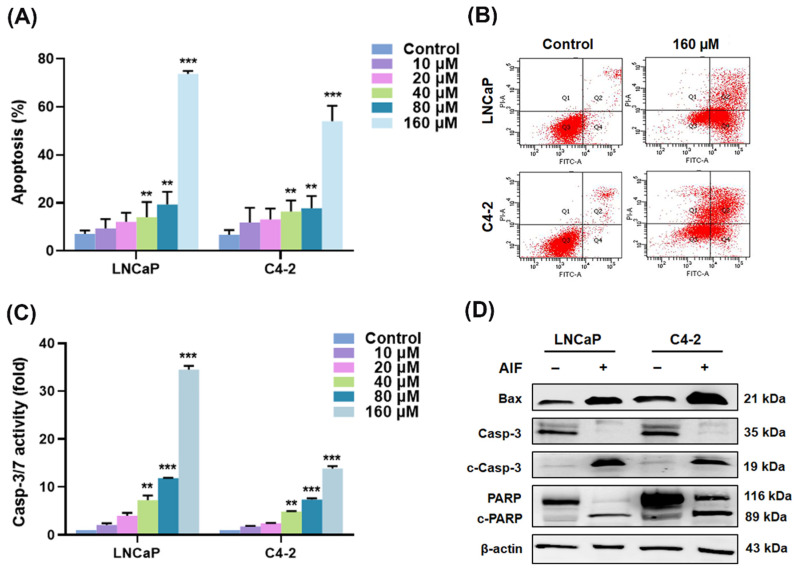
AIF induced apoptosis in PCa cells via the caspase-dependent pathway. (**A**) LNCaP and C4-2 PCa cells were treated with the different concentrations of AIF or vehicle control for 48 h. Subsequently, the apoptotic cells (%) were analyzed with three different studies. (**B**) The representative flow cytometry plots of PCa cells treated with control (1.2% DMSO) or AIF (160 μM) were shown. (**C**) PCa cells were exposed to the various contrations of AIF or control. The activity of caspase-3/7 enzymes were then assayed. The casp-3/7 activity (fold) was assigned as 1.0 in control-treated cells. (**) *p* < 0.01, (***) *p* < 0.001. (**D**) The protein expression levels of pro-apoptotic BAX, caspase-3, and PARP were affectd by AIF in PCa cells determined by Western blot analysis.

Leading to induction of programmed cell death/apoptosis in cancers is one of the most promising efficacies of anti-cancer agents. In addition, some natural compounds have been previously reported to induce apoptosis in cancer cells [33,34,35]. We assumed that AIF could activate apoptosis in PCa cells. To examine whether programmed cell death of PCa caused by AIF was due to the activation of caspase-dependent apoptosis, we first measured the population numbers (%) of apoptotic cells in LNCaP and C4-2 cells after AIF treatment assayed by Annexin V-Fluorescein isothiocyanate (FITC)/propidium iodide (PI) staining with flow cytometry analysis. As shown in Figure 6A, AIF increased the apoptotic cells in a concentration-dependent pattern in both LNCaP and C4-2 cells. Only 7.07 ± 1.19% (LNCaP cells) or 6.70 ± 1.01% (C4-2 cells) of the apoptotic cells were detected with control treatment (Figure 6A). But, the apoptotic cells were substantially elevated in AIF-treated LNCaP (73.77 ± 1.00%; 160 μM) and C4-2 (54.00 ± 5.31%; 160 μM) cells (Figure 6A,B). Subsequently, the enzymatic activities of caspase-3/7 in PCa cells were significantly induced by AIF treatment in a dose-dependent manner (Figure 6C). Moreover, AIF up-regulated pro-apoptotic BAX protein expression and enhanced the protein levels of cleaved caspase-3 and cleaved PARP (89 kDa), which are the activated forms of caspase-3 and PARP, in PCa cells (Figure 6D). The results of the apoptotic analyses collectively suggested that AIF exhibited the attractive efficacy of an anti-cancer drug by induction of caspase-associated apoptosis in PCa cells.

## 4. Discussion

Many natural compounds/products directly extracted from plants, specifically from traditional Chinese medicinal herbs, have been demonstrated to display the substantial effects against cancers [32,36,37]. In this research report, we exclusively evaluated the therapeutic capability of AIF in PCa cells for the first time, which is a pure compound isolated from a traditional Chinese medicinal herb, *Derris eriocarpa*. AIF showed the remarkable anti-cancer effects on both androgen-sensitive LNCaP and castration-resistant C4-2 PCa cells by repressing AR/PSA expression and co-targeting FASN- and HMGCR-mediated fatty acid/lipid and cholesterol biosynthesis leading to inhibition of cell growth, colony formation, migration, and invasion, along with activation of caspase-associated apoptosis.

The activations of the androgen/AR activity and its regulated pathways have been well delineated to induce PCa initiation and promote CRPC progression. Ideally, blockade of the androgen/AR controlled signaling would be able to provide a possible therapeutic strategy to cure PCa malignancy. However, the targeting androgen/AR agents have been employed to treat PCa patients with a short-term survival benefit while the severe adverse effects of these agents lead to CRPC and/or the metastatic status as a result in lethal aggressiveness [38,39,40]. Exploring and finding innovative and alternative drugs that effectively treat this fatal disease are urgently needed to address this ongoing clinical challenge. Notably, reprogram of cellular metabolism is one of the emerging hallmarks in cancers [9]. Particularly, up-regulation of the protein factors regulating fatty acid/lipid/cholesterol biosynthesis to offer crucial precursors and building blocks for cell membrane development and the survival signaling pathway activation in PCa cells has been reported [27,41,42]. Moreover, research articles provided the evidence that a decrease of FASN-mediated or HMGCR-regulated biosynthesis caused the suppression of cell growth, migration, and the survival pathways in cancer cells [20,26,43]. Intriguingly, blockades of lipogenesis and cholesterogenesis controlled by FASN and HMGCR enabled to overcome deadly CRPC progression [20,43]. In this paper, we revealed that AIF not only reduced AR and PSA expression, but also co-suppressed FASN and HMGCR expression in LNCaP and C4-2 cells (Figure 4). Additionally, through inhibition of key regulators controlling lipogenesis and cholesterogenesis, AIF decreased the intracellular fatty acid amounts, the cholesterol levels and lipid droplet accumulation in PCa cells (Figure 5). As expected, targeting the emerging cancer hallmarks and PCa-specific vulnerability genes, such as FASN and HMGCR [44,45], AIF treatment caused the attenuation of PCa cell growth, migration, and invasion. Taken together, these findings suggest that coordinated inhibition of AR, PSA, FASN, and HMGCR expression by AIF could provide a feasible and novel aspect in PCa therapy.

Apoptosis exclusively induced by the caspase pathway has been well recognized as an essential and distinctive mode of programmed cell death that triggered by a series of proteins and enzymes. Among these apoptotic factors, caspase-3 is a major executioner enzyme and a biomarker and plays a key role in regulation of extrinsic and intrinsic apoptosis [46]. Besides, BAX is a pro-apoptotic protein, one of the Bcl-2 family members, and an upstream mediator of caspase-3. Clinically, the efficacy of many FDA-approved anti-cancer agents by attenuating cell growth and the survival pathways is substantially dependent on the activation of apoptosis to eliminate cancer cells [47]. Selectively targeting FASN and lipogenesis have been reported to induce caspase-dependent apoptosis in cancers [48,49,50]. Importantly, our results showed that AIF treatment increased the numbers of the apoptotic cells in PCa cells. Furthermore, caspase-3/7 enzymatic activities, BAX protein as well as the activated (cleaved) forms of caspase-3 and PARP were significantly induced by AIF in LNCaP and C4-2 cells (Figure 6). Collectively, AIF would be able to activate caspase-dependent apoptosis in PCa cells. An additional preclinical study will be warranted to evaluate the effect of AIF on the mouse models bearing xenograft PCa.

## 5. Conclusions

In summary, we endeavored to explore the promising possibility of AIF treatment in PCa aggressiveness. AIF showed a potential to suppress cell growth, anchorage-independent colony formation, migration, and invasion in LNCaP and C4-2 cells. Intriguingly, FASN- and HMGCR-mediated lipogenesis, cholesterogenesis, and AR/PSA were coordinately targeted by AIF in PCa cells. Moreover, AIF led to PCa cell death through activation of caspase-dependent apoptosis. Our data demonstrated that AIF could be applied as a new anti-cancer therapeutic remedy, and provided an alternative opportunity to eradicate PCa.

## Data Availability

Data were contained within the article.

## Supplementary Materials

The following supporting information can be downloaded at: https://www.mdpi.com/article/10.3390/life12111769/s1, Supplementary Figure S1. The chemical structure of AIF. Table S1. The oligonucleotide sequences of the primers used for qPCR analysis.
