# Supplementary material for: Alpinumisoflavone Exhibits the Therapeutic Effect on Prostate Cancer Cells by Repressing AR and Co-Targeting FASN- and HMGCR-Mediated Lipid and Cholesterol Biosynthesis
Source: Life (Basel). 2022 Nov 2;12(11):1769. doi: 10.3390/life12111769 (PMC9698239; doi:10.3390/life12111769)
Supplement: Supplementary file 1 [file life-12-01769-s001.zip › life-1995253-supplementary.pdf]

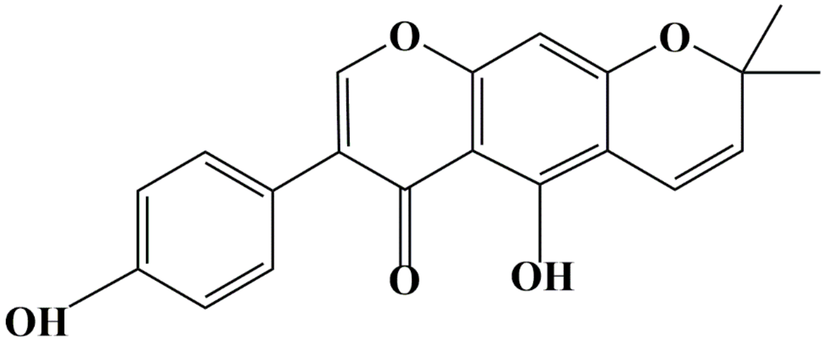

Supplementary Figure S1. The chemical structure of AIF.

Table S1. The oligonucleotide sequences of the primers used for qPCR analysis

Gene	Forward (5’-3’)	Reverse (5’-3’)
AR	GCCTTGCTCTCTAGCCTCAA	GTCGTCCACGTGTAAGTTGC
PSA	GTGTGTGGACCTCCATGTTATT	CCACTCACCTTTCCCCTCAAG
FASN	ACAGCGGGGAATGGGTACT	GACTGGTACAACGAGCGGAT
HMGCR	GTCATTCCAGCCAAGGTTGT	GGGACCACTTGCTTCCATTA
β-actin	AACTGGAACGGTGAAGGTGAC	TGTGGACTTGGGAGAGGACTG
